# Measurement and Comparison of Organic Compound Concentrations in Plasma, Whole Blood, and Dried Blood Spot Samples

**DOI:** 10.3389/fgene.2016.00064

**Published:** 2016-04-21

**Authors:** Stuart A. Batterman, Sergey Chernyak, Feng-Chiao Su

**Affiliations:** Department of Environmental Health Sciences, University of MichiganAnn Arbor, MI, USA

**Keywords:** persistent organic compounds, distribution coefficient, plasma, whole blood, dried blood spots

## Abstract

The preferred sampling medium for measuring human exposures of persistent organic compounds (POPs) is blood, and relevant sample types include whole blood, plasma, and dried blood spots (DBS). Because information regarding the performance and comparability of measurements across these sample types is limited, it is difficult to compare across studies. This study evaluates the performance of POP measurements in plasma, whole blood and DBS, and presents the distribution coefficients needed to convert concentrations among the three sample types. Blood samples were collected from adult volunteers, along with demographic and smoking information, and analyzed by GC/MS for organochlorine pesticides (OCPs), chlorinated hydrocarbons (CHCs), polychlorinated biphenyls (PCBs), and brominated diphenyl ethers (PBDEs). Regression models were used to evaluate the relationships between the sample types and possible effects of personal covariates. Distribution coefficients also were calculated using physically-based models. Across all compounds, concentrations in plasma were consistently the highest; concentrations in whole blood and DBS samples were comparable. Distribution coefficients for plasma to whole blood concentrations ranged from 1.74 to 2.26 for pesticides/CHCs, averaged 1.69 ± 0.06 for the PCBs, and averaged 1.65 ± 0.03 for the PBDEs. Regression models closely fit most chemicals (*R*^2^ > 0.80), and whole blood and DBS samples generally showed very good agreement. Distribution coefficients estimated using biologically-based models were near one and did not explain the observed distribution. Among the study population, median concentrations of several pesticides/CHCs and PBDEs exceeded levels reported in the 2007–2008 National Health and Nutrition Examination Survey, while levels of other OCPs and PBDEs were comparable or lower. Race and smoking status appeared to slightly affect plasma/blood concentration ratios for several POPs. The experimentally-determined distribution coefficients can be used to compare POP exposures across studies using different types of blood-based matrices.

## Introduction

The “gold standard” for assessing environmental exposures to chemicals uses biomonitoring (Sexton et al., 2004), and blood and urine are the most commonly used biological matrices (Aylward et al., 2014). Persistent organic compounds (POPs), e.g., organochlorine pesticides (OCPs) and polychlorinated biphenyls (PCBs), are preferentially measured in blood for several reasons, including their long half-lives (ATSDR, 2000, 2002). Large scale programs monitoring POPs in blood include the U.S. National Health and Nutrition Examination Survey (NHANES), a nationally representative sample that measured OCPs, PCBs and brominated flame retardants (BFRs) levels in serum of adults from 2001 to 2008 (CDC, 2015a), and the German Environmental Surveys, a nationwide population sample that measured OCPs and PCBs in whole blood samples in 1997–1999 and 2003–2006 (Becker et al., 2002; Schulz et al., 2007). There are few if any examples of large biomarker studies of infants and children. However, newborn screening programs aimed at detecting genetic diseases that use dried blood spot (DBS) samples can provide a valuable opportunity to track exposures to environmental pollutants (Olney et al., 2006; Olshan, 2007; Ma et al., 2014; CDC, 2015b). Although DBS samples represent very small volumes of blood (approximately 50 μL per spot), this sampling technique has advantages of simple and safe collection, transport and storage (Jones and Golding, 2009; Lu et al., 2012; Ostler et al., 2014) that may make DBS sampling particularly suitable for large-scale studies and studies of newborns. In additional, some authorities archive these samples, which gives the possibility of retrospective exposure assessments. Environmental pollutants that have been detected in DBSs include several trace metals as well as OCPs, PCBs, and polybrominated diphenyl ethers (PBDEs) (Burse et al., 1997; Lu et al., 2012; Batterman and Chernyak, 2014; Ma et al., 2014). The comparability of methods that measure POPs in different blood matrices, specifically plasma/serum, whole blood, and DBS, has not been reported.

The objectives of this study are to present and evaluate measurement methods for POPs in plasma, whole blood and DBS samples, and to develop conversion or distribution coefficients that can be used for comparisons among studies. An experimental study using a convenience sample and a wide range of POPs was conducted for these purposes.

## Materials and methods

### Participant recruitment and sample collection

A convenience sample of 21 adults was recruited using emails and face-to-face conversations. Inclusion criteria included age greater than 18 years, no underlying health condition or medication use that might make participation risky, ability to provide informed consent, and ability to understand and communicate in English. All participants provided written informed consent. Study protocols were approved by the University of Michigan Institutional Review Board (HUM00090791).

Each participant completed a short questionnaire inquiring about their age, sex, education level, race, and smoking status. Using standard procedures and a professional phlebotomist, each participant provided 10–12 mL venous blood samples drawn from the cubital vein. From this sample, 4 mL was placed into an evacuated Na-heparin/EDTA tubes (4 mL tube, lavender top) for whole blood samples, 6 mL was placed into an evacuated sodium heparin (6 mL tube, green top) for plasma samples, and 10 DBSs were prepared using a 100 μL syringe to apply 50 μL drops to a paper card (Whatman 903 Protein saver card, Sigma-Aldrich, St. Louis, MO, USA), forming circles approximately 12 mm in diameter. DBS samples were labeled, allowed to dry, placed in a paper envelope, and sealed in a polyethylene bag. Green top tubes were centrifuged at 3000 RPM for 20 min to produce approximately 4 mL of plasma. All sample types were stored at −80°C until chemical analysis, which was completed within 3 weeks of sample collection.

### Analyses and quality assurance

Blood samples were analyzed for 54 persistent organic compounds: 19 organochlorine pesticides, 2 other chlorinated hydrocarbons (CHCs), 12 polychlorinated biphenyls (PCBs), 20 brominated diphenyl ethers (PBDEs), and tetrabromobisphenol A (TBBA) (Supplemental Table S1). The target compounds were selected as they have been widely used, have long half-lives in the environment and humans, and have been associated with adverse health effects, e.g., cancer and neurological disorders (ATSDR, 2000, 2002, 2004). These compounds have a range of chemical structure and have been used as target compounds in other projects.

Sample preparation and analysis procedures followed Batterman and Chernyak (2014) and Schantz et al. (2013). DBS punches (12 mm dia) and 1 mL subsamples of blood and plasma were spiked with 15 μL of surrogate solution, and sonicated for 20 min after adding 1 mL hydrochloric acid (6M), and sonicated a second time for 20 min after adding 6 mL of ethanol/isopropanol (1:1). Samples were extracted twice using 6 mL hexane/methyl-t-butyl ether (MTBE, 1:1). After rotating for 2 min, the organic layer was transferred to a new tube, with 4 mL 1% potassium chloride, and the sample was re-extracted with 3 mL hexane/MTBE (1:1). The organic layers were combined, and the solvent volume was reduced to 1 mL under a gentle N_2_ flow. Next, the concentrated sample was eluted through a sulfuric acid/silica gel column (Akros Corp, 60–200 μm; 1 g silica/33% H_2_SO${}_{4}^{-}$ 0.1 g pure silica). Plasma did not require this sulfuric acid clean-up; identical results were obtained for plasma samples with and without additional sulfuric acid clean-up. Analytes were eluted from the column using 8 mL of a hexane/DCM mixture (1:1), and evaporated under N_2_ to 0.5 mL. The final extract was prepared by adding 0.5 mL n-nonane, and then evaporating under N_2_ to a final volume of 250 μL.

Analyses used a gas chromatograph/mass spectrometer (GC/MS; 6890/5973, Agilent Industries, Palo Alto, CA, USA), a DB-5 column for separation (30 m, 0.25 mm dia., film thickness 0.25 μm; J&W Scientific, Folsom, CA, USA), and electron capture negative chemical ionization (NCI SIM) detection. Helium was used as the carrier gas and methane as the mass spectrometry reagent gas. The helium flow rate was 0.7 mL min^−1^, pressure was 5.43 psi, and the average velocity was 31 cm s^−1^. The injection syringe was 10 μL, and the injection volume was 2 μL. Separate runs were used for pesticides and other chlorinated hydrocarbons, tri- through octa-BFRs, nona- and deca-BFRs, and PCBs; these required from 34.5 min (BFRs) to 69.5 min (pesticides). GC temperature programs and MS settings were optimized for the three compound groups (Batterman and Chernyak, 2014). For PCBs, the initial oven temperature setting was 80°C, which was held for 1 min, then ramped at 20°C/min to 150°C, then ramped at 2°C/min to 250°C, held for 4 min, ramped at 30°C/min to 300°C, and finally held for 6 min. For pesticides, the initial temperature was 80°C, held for 1 min, ramped at 20°C/min to 150°C, ramped at 2°C/min to 250°C, held for 4 min, ramped at 10°C/min to 300°C, and then held for 6 min. For tetra- through octa-homologs of PBDEs, the temperature started at 80°C, held for 2 min, and then ramped at 10°C/min to 300°C. For nona- and deca-homologs of PBDEs, the initial temperature was 80°C, held for 2 min, ramped at 50°C/min to 300°C, and then held for 55 min. Ions and retention times are presented elsewhere (Batterman and Chernyak, 2014).

Calibration standards used certified materials (persistent pesticides, PCB and PBDE calibration solutions, Cambridge Isotope Laboratories, Tewksbury, MA, USA) prepared at concentrations of 0.01 and 0.05 (for DBS), 0.1, 0.5, 1, 10, and 50 ng mL^−1^ (all sample types). This range was selected to span the range expected in human samples. Sample replicates, performed in all cases, were averaged. Other calibration protocols are described elsewhere (Batterman and Chernyak, 2014).

Instrumental detection limits (IDLs) were defined as the amount of analyte for which the signal-to-noise ratio of the peak was equal to three, based on analyses of low concentration samples. The IDLs were determined by preparing and analyzing composite samples of plasma and whole blood (collected from 5 volunteers), and assuming a final extraction volume of 250 μL and plasma/serum volume of 1 mL. For plasma and whole blood samples, IDLs for pesticides/CHCs, PCBs and BFRs ranged from 5 to 30, 0.1 to 0.9, and 0.1 to 30 ng L^−1^, respectively. For DBS samples, IDLs for pesticides, PCBs and BFRs were higher, 10–120, 0.8–22, and 8–200 ng L^−1^, respectively. These IDLs are appropriate for the distribution coefficients derived for this study.

QA procedures are described in Batterman and Chernyak (2014). In brief, a minimum of two (for plasma/blood) or three (for DBS) replicates were analyzed; these had high agreement. Internal standards (IS), quantified in each run, used labeled mixtures. Each set of analyses used quality control (QC) samples that were processed in parallel with samples to check drift (standard solution ran at the beginning and end of each batch), blank contamination (including laboratory and field blanks), linearity, spike recovery, and accuracy using a standard reference material (SRM 1957, Organic Contaminants in Non-Fortified Human Serum, NIST, Gaithersburg MD, USA). Results were accepted only when replicate values varied by less than 10%, linearity defined by R^2^ exceeded 0.999, and measurements of the SRM were within 20% of certified value. All spike recoveries were in the range of 76–102%. Levels of blank contamination in standards and in processing materials were below IDLs, although trace levels of β-hexachlorocyclohexane, *trans*-chlordane, *cis*-chlordane, *trans*-nonachlor, p,p'-DDE, PCBs-132/153, 138/163, 202, 180, and PBDEs-47 and 99 were found; these levels were well below concentrations in actual samples by 15-fold for DDE, 600 to 6000-fold for other pesticides, 10–70-fold for PBDEs, 7-fold for PCB-202, and 200–2000-fold for the other PCBs.

### Data analyses

Compounds detected in fewer than 25% of the samples above IDL were excluded from further analysis (Supplemental Table S1). Measurements below the IDL were replaced by ½ IDL. Descriptive analyses, including central tendency and dispersion, were calculated for the demographic and biomarker data. The normality of measurements was evaluated using Kolmogorov-Smirnov tests. Associations between pairs of biomarkers were examined using Spearman rank-order correlations. Diagnostics used to detect potential outliers included the use of scatterplots between each sample types, and modified Bland-Altman plots. In a few cases (described below), data points were removed when measurements between the sample types showed significant disagreements.

Biomarker levels were compared to levels reported in NHANES 2007–2008, the latest US national-level study that measured POPs. NHANES used pooled serum samples (*n* = 264 pooled samples), each based on contributions from 8 individuals (CDC, 2015a). A total of 24 POPs were common to both NHANES and this study, of which 16 had detection frequencies exceeding 25%. Since NHANES used a weighted pooled-sample design, appropriate sample weights were applied when estimating concentrations. Differences in POP concentrations between this study and NHANES were tested using *t*-tests (for weighted samples) and Wilcoxon-Mann-Whitney tests.

### Distribution models

The experimental distribution coefficient for each POP was estimated as the fitted coefficient (β) in linear models that regressed concentrations in plasma (or blood) against concentrations in whole blood, e.g., K_p∕b_ is the expected ratio of concentrations in plasma to those in blood. The goodness-of-fit (GOF) was examined using R^2^ for models with intercepts, and the estimated R${}_{\text{est}}^{2}$ for models without intercepts, where R${}_{\text{est}}^{2}$ = 1–[(error sum of squares for without-intercept models)/(corrected total sum of squares from with-intercept models)].

Theoretical estimate of distribution coefficients for each POP were calculated using a physically-based model that accounted for chemical solubility in lipid and water, as well as the lipid and water content in plasma and whole blood (Poulin and Krishnan, 1995):

(1) $$\begin{array}{l} {\text{K}}_{\text{t},\text{p/b}}=\left({\text{S}}_{\text{o}}{\text{N}}_{\text{p}}\text{+}{\text{S}}_{\text{w}}\text{0}.{\text{7P}}_{\text{p}}\text{+}{\text{S}}_{\text{o}}\text{0}.{\text{3P}}_{\text{p}}\text{+}{\text{S}}_{\text{w}}{\text{W}}_{\text{p}}\right)/\\ \;\left({\text{S}}_{\text{o}}{\text{N}}_{\text{b}}\text{+}{\text{S}}_{\text{w}}\text{0}.{\text{7P}}_{\text{b}}\text{+}{\text{S}}_{\text{o}}\text{0}.{\text{3P}}_{\text{b}}\text{+}{\text{S}}_{\text{w}}{\text{W}}_{\text{b}}\right) \end{array}$$

where S_o_ = solubility of chemical in n-octanol (mg L^−1^), N_p_ = neutral lipid content of plasma, S_w_ = solubility of chemical in water (mg L^−1^), P_p_ = phospholipid content of plasma (Phillips et al., 1989), W_p_ = water content of plasma (Faye and Payne, 1986), N_b_ = neutral lipid content of blood (Poulin and Krishnan, 1995), P_b_ = phospholipid content of blood (Poulin and Krishnan, 1995), and W_b_ = water content of blood (Poulin and Krishnan, 1995). The solubility in n-octanol was estimated as the product of the chemical's solubility in water and its octanol-water distribution coefficient (K_ow_), both of which were obtained from EPI Suite Version 4.11 (USEPA, 2015).

Possible effects of demographic and smoking variables on distribution coefficients were investigated by regressing the ratio of concentrations measured in plasma and whole blood (using individual samples) against demographic and smoking variables.

Statistical analyses and model fitting were performed using SAS 9.2 (SAS Institute, Cary, NC, USA).

## Results and discussion

### Descriptive analyses and data cleaning

Participants averaged 48 years in age (range: 29–65 years; *n* = 21). Slightly over half (57%, *n* = 12) were male, 76% (*n* = 16) were white, and 67% (*n* = 14) had graduate degrees (Supplemental Table S2). Most (76%, *n* = 16) were lifetime non-smokers; one (5%) was a current smoker.

After reviewing the data, two observations were considered outliers and were removed from further analysis to improve reliability. Pentachlorobenzene and β-hexachlorocyclohexane (β-HCH) each had a single outlier (Supplemental Figure S1), which when removed increased the fit of the distribution models and improved agreement with distribution coefficients of the other pesticides. α-HCH had slightly lower fit (*R*^2^ = 0.76) than other POPs, probably due to low detection frequencies in plasma (48%) and whole blood (33%) samples, but no outliers were identified.

**Figure 1:**
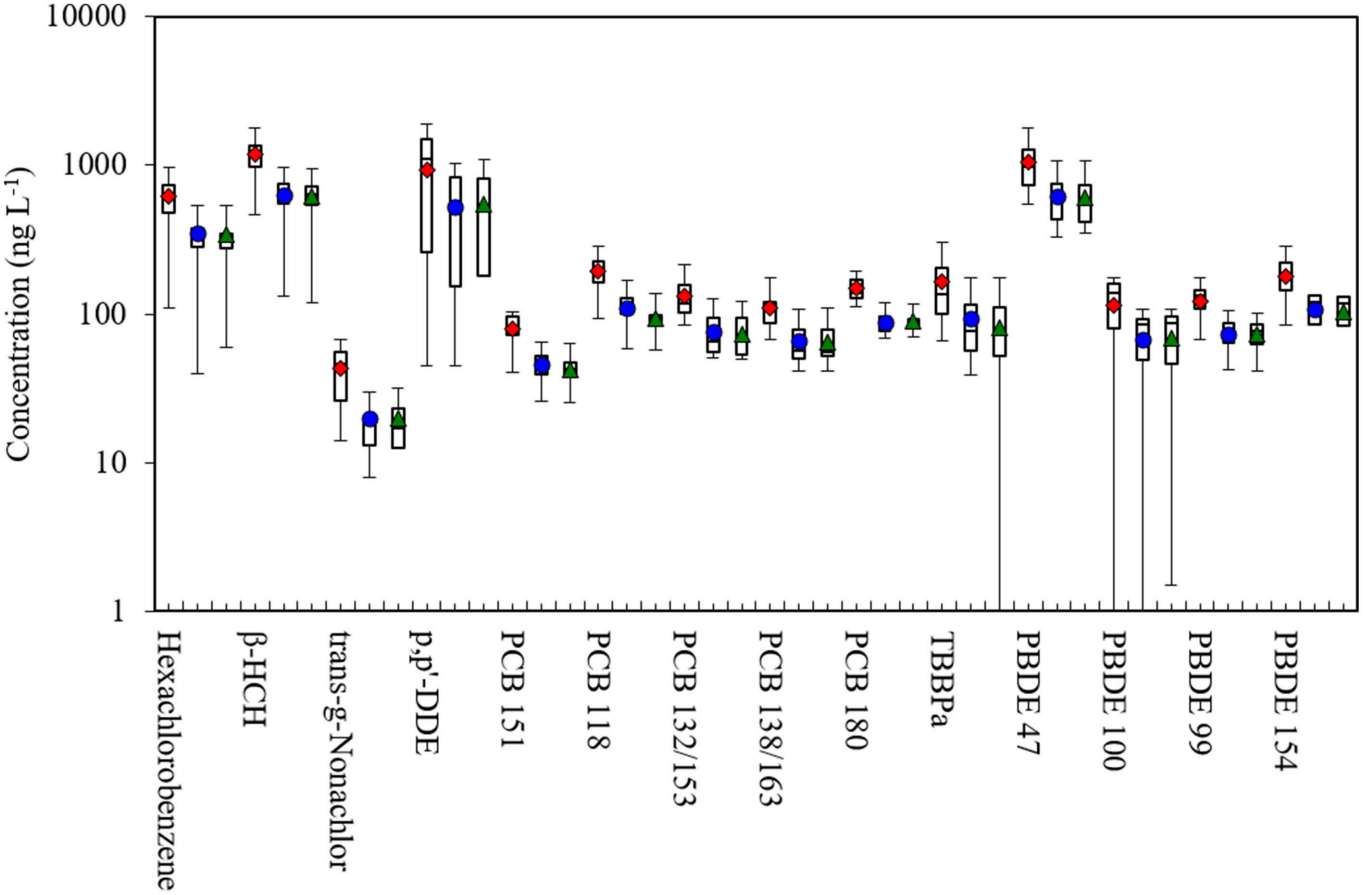
**Box plots of POP concentrations in plasma, whole blood and dried blood spot (DBS) samples in this study**. Shows 5th, 25th, 50th, 75th, and 95th percentiles and average. Red diamond: plasma; blue dot: whole blood; green triangle: DBS. β-HCH, beta-hexachlorocyclohexane; PCB, polychlorinated biphenyl; TBBPa, tetrabromobisphenol A; PBDE, polybromodiphenyl ether.

Of the 54 target compounds, detection frequencies exceeded 25% for 33 compounds in plasma, 32 compounds in whole blood, and 14 compounds in DBS samples (Supplemental Table S1). For all compounds, concentrations in plasma exceeded levels in whole blood, and blood and DBS samples had similar concentrations (Figure 1). The limited sample volume of the DBS samples resulted in higher detection limits, which limited the number of analytes that could be detected using the GC/MS. Among pesticides/CHCs, β-HCH had the highest median concentration (1243, 668, and 641 ng L^−1^ in plasma, whole blood and DBS samples, respectively); PCB-118 was the highest among PCBs (196, 104, and 95 ng L^−1^), and PBDE-47 was the highest among flame retardants (998, 593, and 546 ng L^−1^). The concentration data fit normal distributions for 23 of the compounds in plasma, 17 of the compounds in whole blood, and 8 of the compounds in DBS samples. Other statistics by sample type are shown in Table 1.

**Table 1:** **Summary statistics of POP concentrations (ng L^−1^) by sample type (*n* = 21)**.

**Chemical**	**Plasma**						**Whole blood**						**Dried blood spot**					

	**Mean**	**SD**	**Min**	**50th**	**Max**	***p*-value**	**Mean**	**SD**	**Min**	**50th**	**Max**	***p*-value**	**Mean**	**SD**	**Min**	**50th**	**Max**	***p*-value**
**Pesticides** + **CHC**																		
Pentachlorobenzene	124.1	60.3	15.0	113.5	270.0	>0.15	68.2	22.0	36.0	70.0	100.0	>0.15	–	–	–	–	–	–
α-HCH	13.2	10.7	5.0	5.0	36.7	< 0.01	7.6	4.4	5.0	5.0	20.0	< 0.01	–	–	–	–	–	–
Hexachlorobenzene	618.1	308.9	5.0	600.0	1560.0	0.136	350.8	183.2	5.0	350.0	960.0	< 0.01	336.8	178.1	25.0	326.3	936.4	< 0.01
β-HCH	1202.4	423.0	227.6	1242.5	1935.0	>0.15	654.7	242.6	132.1	667.7	1130.0	0.127	604.5	273.5	30.0	641.0	1073.9	0.067
Dachtal	130.7	103.6	5.0	130.0	420.0	>0.15	74.7	55.5	5.0	70.0	200.0	>0.15	–	–	–	–	–	–
b-Heptachlorepoxide	16.4	16.3	7.5	7.5	70.0	< 0.01	–	–	–	–	–	–	–	–	–	–	–	–
*trans*-Chlordane	45.5	17.0	20.0	49.0	72.0	>0.15	20.2	6.9	5.0	20.0	30.0	< 0.01	–	–	–	–	–	–
*cis*-Chlordane	35.7	22.0	5.0	30.0	80.0	0.044	17.3	8.9	5.0	15.7	40.0	0.046	–	–	–	–	–	–
*trans*-Nonachlor	43.2	23.5	2.5	46.0	113.0	>0.15	19.7	10.5	2.5	20.0	54.0	< 0.01	17.2	12.7	6.0	17.0	56.0	< 0.01
p,p'-DDE	956.2	751.6	15.0	1130.0	2480.0	>0.15	529.8	401.0	15.0	560.0	1354.0	>0.15	510.4	390.8	50.0	546.0	1273.0	>0.15
*cis*-Nonachlor	37.7	15.4	5.0	38.0	70.0	>0.15	17.8	9.3	5.0	20.0	37.2	< 0.01	–	–	–	–	–	–
**PCBs**																		
PCB-110	1.23	0.31	0.81	1.14	2.00	< 0.01	0.75	0.20	0.54	0.68	1.25	< 0.01	–	–	–	–	–	–
PCB-151	80.22	23.95	15.45	81.04	126.48	>0.15	45.83	13.29	9.34	44.67	72.54	0.148	42.45	13.91	0.40	42.45	64.34	>0.15
PCB-135/144	15.78	4.41	6.43	16.89	22.10	>0.15	9.26	2.47	4.22	9.69	13.56	>0.15	–	–	–	–	–	–
PCB-118	196.61	63.47	44.34	196.02	312.80	>0.15	111.48	36.33	28.14	104.37	184.83	< 0.01	92.74	28.95	0.40	95.39	145.33	< 0.01
PCB-132/153	133.12	44.58	47.11	119.65	225.70	0.124	76.51	28.11	28.65	67.98	133.27	0.044	73.66	27.63	26.58	64.93	127.55	0.017
PCB-138/163	109.35	38.81	38.34	103.73	189.87	>0.15	65.91	24.11	23.56	56.32	118.43	< 0.01	65.01	24.26	27.57	55.05	113.62	< 0.01
PCB-175	18.70	7.96	5.87	19.52	33.55	0.038	11.04	4.62	3.35	11.65	19.99	>0.15	–	–	–	–	–	–
PCB-174	1.92	0.72	0.45	2.04	3.22	>0.15	1.20	0.45	0.35	1.24	2.19	>0.15	–	–	–	–	–	–
PCB-202	1.95	0.78	0.33	2.09	3.04	>0.15	1.13	0.38	0.27	1.26	1.78	< 0.01	–	–	–	–	–	–
PCB-180	147.83	26.62	111.54	139.36	198.32	0.032	88.50	15.45	61.12	85.15	119.64	>0.15	88.91	15.51	63.60	85.27	124.48	>0.15
PCB-170/190	9.02	3.75	0.40	9.23	15.30	>0.15	5.08	2.28	0.40	4.82	9.86	0.067	–	–	–	–	–	–
PCB-198	6.45	2.23	2.69	6.19	10.69	>0.15	4.07	1.26	1.44	4.24	6.53	>0.15	–	–	–	–	–	–
**BFRs**																		
TBBPa	159.14	85.73	65.65	133.53	349.02	0.024	90.16	49.81	33.23	75.34	201.34	0.021	81.39	55.62	6.00	72.53	193.45	>0.15
PBDE-28	21.19	18.42	3.50	17.44	64.89	< 0.01	13.53	11.16	3.50	10.86	40.76	< 0.01	20.00	0.00	20.00	20.00	20.00	
PBDE-75	48.86	54.63	2.50	37.28	189.66	< 0.01	29.30	32.15	2.50	23.41	112.45	< 0.01	20.00	0.00	20.00	20.00	20.00	
PBDE-47	1052.28	381.16	454.56	997.89	1858.00	>0.15	628.16	229.13	218.56	593.43	1099.61	>0.15	605.60	222.96	227.34	546.23	1076.23	>0.15
PBDE-66	191.75	120.31	2.50	179.32	377.66	>0.15	115.29	71.00	2.50	107.76	224.71	>0.15	–	–	–	–	–	–
PBDE-100	113.83	59.26	4.00	139.49	184.47	0.038	68.72	35.30	4.00	85.87	111.82	0.017	70.97	31.91	20.00	86.06	116.63	0.021
PBDE-99	125.21	36.99	44.66	135.66	181.63	>0.15	75.67	22.53	25.76	81.27	109.88	>0.15	72.95	21.48	24.47	77.42	104.21	>0.15
PBDE-85	89.16	75.25	0.25	96.74	287.21	0.128	54.13	44.86	0.25	59.69	169.29	0.107	–	–	–	–	–	–
PBDE-154	180.79	72.94	0.05	176.19	295.48	>0.15	106.68	46.77	0.05	119.42	175.14	>0.15	103.91	44.08	10.00	117.67	168.87	>0.15
PBDE-153	9.54	6.99	0.05	8.65	23.13	>0.15	6.01	4.15	0.05	5.55	14.37	>0.15	–	–	–	–	–	–

*β-HCH, beta-hexachlorocyclohexane; PCB, polychlorinated biphenyl; BFR, brominated flame retardant; TBBPA, tetrabromobisphenol A; PBDE, polybromodiphenyl ether; SD, standard deviation; p-value, p-values of Kolmogorov-Smirnov tests for normality test*.

Concentrations of POPs within a chemical group included a number of compound pairs that were moderately or highly correlated (Supplemental Table S3). Considering concentrations in plasma, for example, one-fifth of pesticide/CHC pairs had Spearman correlation coefficients from 0.44 to 0.74; the highest correlations were between β-HCH and *trans*-chlordane (*r* = 0.74), and between *cis*-nonachlor and *trans*-nonachlor (*r* = 0.66). PCB congeners showed the strongest correlation (most congener pairs above 0.7) with the exception of pairs involving PCB-110. Among the BFRs, PBDE-66, 85, 99, and 100 were highly correlated, reflecting their common source in PBDE technical mixtures. Across the chemical groups, a few pairs of POPs were moderately correlated, either positively or negatively. Overall, measurements of the different POP types across the volunteers had only limited correlation, which is expected given their different exposure sources, however, the sample size is too limited to draw conclusions regarding possible explanatory factors.

**Figure 2:**
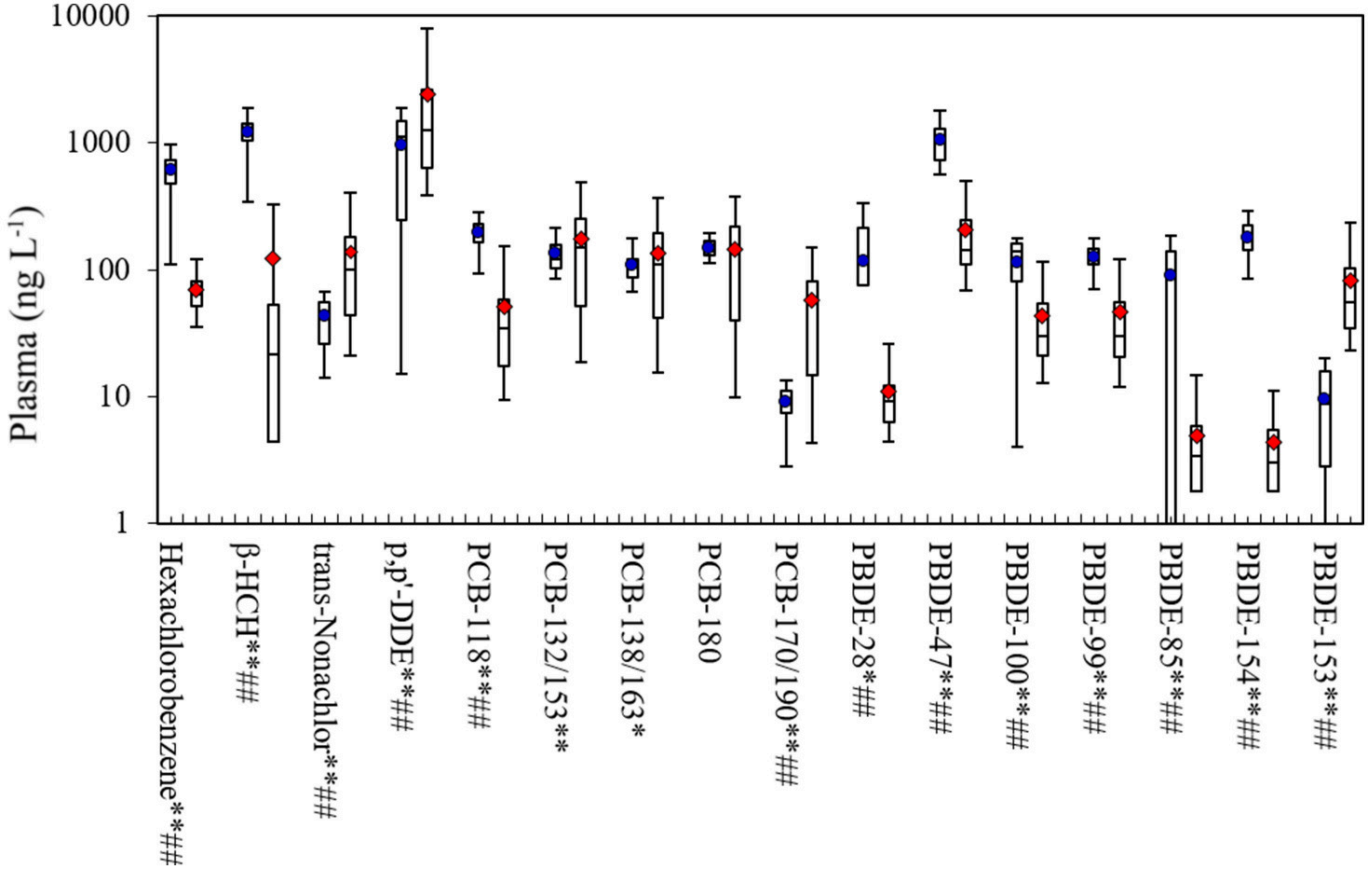
**Box plots of POP concentrations in plasma in this study and NHANES 2007–2008**. Shows 5th, 25th, 50th, 75th and 95th percentiles and average. Average for the current study shown in dot for this study, and diamond for the NHANES 2007–2008; ^**^p < 0.01 and ^*^p < 0.05 for *t*-tests; ^*##*^*p*-value < 0.01 and ^#^*p*-value < 0.05 for Wilcoxon-Mann-Whitney tests. Sample size for this study was 21, and 264 (pooled samples) for NHANES. β-HCH, beta-hexachlorocyclohexane; PCB, polychlorinated biphenyl; BFR, brominated flame retardant; TBBPA, tetrabromobisphenol A; PBDE, polybromodiphenyl ether.

Figure 2 compares our measurements of POPs in plasma to levels in the NHANES 2007–2008, which are often used as U.S. reference values (CDC, 2015a). For pesticides/CHCs and considering medians, we found much higher levels of hexachlorobenzene (600 vs. 63 ng L^−1^) and β-HCH (1243 vs. 21 ng L^−1^), lower levels of *trans*-nonachlor (46 vs. 101 ng L^−1^), and comparable levels of p,p'-DDE (1130 vs. 1256 ng L^−1^). Concentrations of most PCB congeners were similar, including PCBs-132/153, 138/163, and 180 (*p* > 0.05 for Wilcoxon-Mann-Whitney tests), although we had higher levels of PCB-118 and lower levels of PCB-170/190. For the PBDEs, we found higher concentrations than NHANES with the exception of PBDE-153. Considering means, our samples differed from NHANES for all POPs except for PCB-180 (*p* < 0.05; *t*-tests). Many factors might explain these differences, including the 7 year gap between the studies (2014 vs. 2007–2008) and temporal trends in POP use; regional differences associated with POP use, environmental concentrations and intake rates (e.g., due to differences in POP fate and diets, particularly fish consumption); differences in the study populations (e.g., small convenience vs. race-sex-age matched samples); and differences in study design (e.g., individual vs. pooled samples). These and potentially other factors make it unlikely that our convenience sample was representative of US adults or the NHANES measurements, however, this should not affect measurement performance or distribution results, discussed next.

### Distribution coefficients

Regression models with and without intercepts gave very similar estimates of K_p∕b_. Results of models without intercepts are shown in Table 2 (models using intercepts are shown in Supplemental Table S4). The experimental values of K_p∕b_ ranged from 1.74 to 2.26 for pesticides/CHCs, and averaged 1.69 ± 0.06 for PCBs, 1.65 ± 0.03 for PBDEs, and 1.75 for tetrabromobisphenol A. The distribution coefficients did not vary by concentration as shown by modified Bland-Altman plots (Supplemental Figure S2). The coefficient of variation, which represents the variation among the 21 participants, was typically within 3%, and model fit was high (*R*^2^ > 0.80) in most cases. Fit was especially high for BFRs (*R*^2^ > 0.91), reflecting the strong GC/MS response and good separation of these compounds in all sample matrices.

**Table 2:** **Experimental distribution coefficients for plasma, whole blood, and DBS samples (***n*** = 21)**.

**Chemicals**	**y** = **plasma, x** = **whole blood**			**y** = **plasma, x** = **DBS**			**y** = **whole blood, x** = **DBS**		

	***R*^2^**	**Coefficient**		***R*^2^**	**Coefficient**		***R*^2^**	**Coefficient**	

		**Estimate**	**SE**		**Estimate**	**SE**		**Estimate**	**SE**
**Pesticides** + **CHC**									
Pentachlorobenzene	0.73	1.92	0.10	–	–	–	–	–	–
α-HCH	0.76	1.83	0.13	–	–	–	–	–	–
Hexachlorobenzene	0.96	1.74	0.03	0.93	1.80	0.05	0.99	1.04	0.01
β-HCH	1.00	1.82	0.02	0.97	1.87	0.02	0.99	1.03	0.01
Dachtal	0.94	1.77	0.06	–	–	–	–	–	–
*trans*-Chlordane	0.89	2.26	0.06	–	–	–	–	–	–
*cis*-Chlordane	0.93	2.13	0.07	–	–	–	–	–	–
*trans*-Nonachlor	0.91	2.18	0.07	0.50	2.18	0.17	0.63	1.00	0.07
p,p'-DDE	0.96	1.82	0.05	0.93	1.87	0.07	0.95	1.02	0.03
*cis*-Nonachlor	0.80	2.01	0.08	–	–	–	–	–	–
**PCBs**									
PCB-110	0.86	1.63	0.03	–	–	–	–	–	–
PCB-151	0.91	1.75	0.03	0.79	1.86	0.05	0.91	1.06	0.02
PCB-135/144	0.93	1.71	0.03	**-**	**-**	**-**	**-**	**-**	**-**
PCB-118	0.91	1.76	0.03	0.51	2.08	0.10	0.47	1.18	0.06
PCB-132/153	0.95	1.72	0.03	0.94	1.78	0.03	1.00	1.04	0.00
PCB-138/163	0.96	1.65	0.02	0.93	1.67	0.03	0.99	1.01	0.01
PCB-175	0.91	1.69	0.04	–	–	–	–	–	–
PCB-174	0.85	1.59	0.05	–	–	–	–	–	–
PCB-202	0.88	1.74	0.05	–	–	–	–	–	–
PCB-180	0.80	1.67	0.03	0.75	1.66	0.03	0.97	1.00	0.01
PCB-170/190	0.88	1.74	0.05	–	–	–	–	–	–
PCB-198	0.80	1.58	0.05	–	–	–	–	–	–
**BFRs**									
TBBPa	1.00	1.75	0.01	0.89	1.82	0.06	0.90	1.04	0.04
PBDE-28	0.99	1.60	0.03	–	–	–	–	–	–
PBDE-75	0.99	1.68	0.03	–	–	–	–	–	–
PBDE-47	0.99	1.67	0.01	0.97	1.73	0.02	0.98	1.04	0.01
PBDE-66	1.00	1.67	0.01	–	–	–	–	–	–
PBDE-100	1.00	1.66	0.01	0.95	1.64	0.04	0.96	0.99	0.02
PBDE-99	1.00	1.65	0.01	0.99	1.72	0.01	0.99	1.04	0.01
PBDE-85	1.00	1.66	0.01	–	–	–	–	–	–
PBDE-154	0.91	1.66	0.04	0.89	1.71	0.05	1.00	1.03	0.01
PBDE-153	0.97	1.61	0.03	–	–	–	–	–	–

*Based on linear regression models without intercepts. R^2^ for the no-intercept model was estimated by 1-[(error sums of squares for without intercept models)/(corrected total sums of squares from with intercept modes)]. DBS, dried blood spot; β-HCH, beta-hexachlorocyclohexane; PCB, polychlorinated biphenyl; BFR, brominated flame retardant; TBBPA, tetrabromobisphenol A; PBDE, polybromodiphenyl ether; SE, standard error*.

The literature contains biomarker measurements from four types of blood samples. Whole blood samples typically are collected in tubes containing an anticoagulant (commonly EDTA). DBS samples represent whole blood without EDTA (or other) treatments. Serum samples (not analyzed in the present study) are collected in (clean) tubes, allowed to clot (coagulate) for 30–45 min at room temperature, centrifuged, and pipetted. (The cell fraction or pellet is typically discarded). Plasma is obtained from blood collected in tubes containing an anticoagulant; the sample is centrifuged as soon as practicable following collection. Plasma, the liquid fraction above the denser fraction (pellet), accounts for roughly half of the whole blood weight.

Blood is a complex matrix containing cells (including red blood cells or erythrocytes, 42–45% wt; white blood cells or leukocytes, 7%; and platelets or thrombocytes), other cellular material, lipids (including neutral lipids, 0.44%; and phospholipids at 0.21%), clotting factor proteins, electrolytes, glucose, nutrients, hormones, water, and other substances. Lipophilic compounds, like most organochlorine compounds, will be entirely or largely absorbed or adsorbed to the lipid and protein components of blood, and thus concentrations in cellular material is expected to be low. If the compound was present only in plasma (or serum) and none was in the centrifuged fraction, then plasma (serum) would have concentrations about 1.8 times higher whole blood (fraction = 1/non-plasma fraction; assumed plasma weight fraction = 0.55). Experimental values of K_p∕b_ ranged from 1.58 (PBDEs) to 2.26 (chlordanes), suggesting that plasma contains 67 (PBDEs) to 100% (chlordanes) of the compound mass present in the blood sample (the plasma content involved in partitioning was reduced from 55 to 45% to fit experimental results). A formal distribution model is discussed in the subsequent section.

Concentrations in DBS and whole blood samples had very high agreement, e.g., DBS/whole blood ratios ranged from 0.99 to 1.18, and distribution coefficients did not vary from one for half of the POPs (*p* < 0.05). Linearity was high (*R*^2^ > 0.90) for most compounds, but somewhat lower for *trans*-nonachlor (*R*^2^ = 0.63) and PCB-118 (0.47), probably due to chromatographic issues (these peaks were poorly separated). Given the greater precision and higher detection frequency of measurements in whole blood compared to DBS, the use of the plasma/whole blood distribution coefficient is preferred over the plasma/DBS coefficient. Still, K_p∕b_ and K_p∕d_ values did not differ in most cases, although the *R*^2^ for K_p∕d_ models were lower. Again, *trans*-nonachlor and PCB-118 had lower fit (*R*^2^ = 0.50 and 0.51, respectively).

Overall, while we cannot determine whether our sample is representative, we saw no effects due to concentration and only minor effects of other factors (discussed below), increasing our confidence in the distribution coefficients reported in Table 2. Several studies using human blood, plasma, and serum have shown comparable results, although each study typically focuses on only a few compounds. A regression analysis showed that concentrations in plasma were elevated over blood by a factor of 1.32 for PCB153 and 1.35 for total PCBs (Sandau et al., 2000); perfluorinated chemicals, which can behave differently, showed slightly lower ratios, about 1.2–1.4 (Kärrman et al., 2006); and a ratio of 1.5 was shown for PBDE-209 between serum and whole blood (Leslie et al., 2007).

### Modeled distribution coefficients

Distribution coefficients K_t_,_p∕b_, estimated using Equation (1), ranged from 1.048 to 1.058 and showed little variation across the chemicals. All values were well below experimental results (1.58 < K_p∕b_ < 2.26; Supplemental Table S5). Predicted values were near unity since the neutral lipid content and phospholipid content of plasma and whole blood are similar. All of the target POPs are highly lipophilic (K_ow_ from 2 × 10^4^ to 4 × 10^8^, Supplemental Table S5), and no relationship was seen between K_ow_ and K_p∕b_ and thus, despite the large range of K_ow_, all of the POPs were sufficiently lipophilic to minimize differences in the plasma/whole blood distribution by this variable. Equation (1) was designed to estimate distribution coefficients for various tissues and blood, reflecting solubility differences between the two matrices (Poulin and Krishnan, 1995). Its key drivers, the neutral lipid and phospholipid contents, can differ substantially between tissues and whole blood and produce large differences. For example, considering pentachlorobenzene and comparing liver and whole blood compartments with neutral lipid contents of 0.0281 and 0.0044, respectively; phospholipid contents of 0.0389 and 0.0021; and water contents of 0.72 vs. 0.80 (Poulin and Krishnan, 1995), the liver/whole blood distribution coefficient is 9.37, nearly 9-fold higher than the plasma/whole blood distribution coefficient of 1.048. The higher levels of POPs observed in plasma suggest that neither the phospholipid nor the neutral lipid contents of blood and plasma govern the distribution of POPs. While accounting for lipid and water content, Equation (1) does not explain effects of protein binding, metabolic process of chemicals, complex cellular matrices, and other factors that may explain differences (Poulin and Krishnan, 1995). Thus, experimentally-derived values of distribution coefficients should be used.

### Effects of demographics and smoking status on plasma-blood ratios of chemical concentrations

Sex, race, and smoking status showed small effects on the plasma/blood distribution of several chemicals (Table 3). Males had higher K_p∕b_ for p,p'-DDE and PBDE-153 (by 0.63 ± 0.20 and 0.31 ± 0.11, respectively). White participants had slightly lower ratios for *cis*-nonachlor, PBDE-47 and 154 (0.12 ± 0.05 to 0.82 ± 0.38), and higher ratios for PCB-151, 118, 132/153, 175, 174, 202, 170/190, PBDE-66 and 100 (−0.33 ± 0.14 to −0.16 ± 0.08) compared to other races (African Americans and Asians). Former or current smokers had lower ratios for hexachlorobenzene, PCB-151, 118 and 132/153 (−0.35 ± 0.14 to −0.16 ± 0.08), and higher ratios for *cis*-chlordane and *trans*-nonachlor (0.52 ± 0.20 and 0.42 ± 0.21, respectively). Even though effects of demographic and smoking status variable appear genuine, most differences were within 20% of the mean, suggesting that impacts from demographic and smoking variables were small.

**Table 3:** **Coefficients of linear regression models examining effects of demographic variables and smoking status on ratio of POP concentrations in plasma and whole blood (***n*** = 21)**.

**Chemicals**	**Age (year)**			**Sex (Ref = female)**			**Race (Ref = white)**			**Educational level (Ref** = ≥ **master's degree)**			**Smoking status (Ref** = non-smoker)		
	**Estimate**	**SE**	***p*-value**	**Estimate**	**SE**	***p*-value**	**Estimate**	**SE**	***p*-value**	**Estimate**	**SE**	***p*-value**	**Estimate**	**SE**	***p*-value**
**Pesticides** + **CHC**															
Pentachlorobenzene	−0.006	0.010	0.533	0.358	0.214	0.094	−0.112	0.257	0.664	−0.260	0.237	0.274	0.373	0.267	0.162
α-HCH	0.013	0.015	0.369	0.496	0.315	0.116	−0.399	0.377	0.291	−0.153	0.348	0.660	0.455	0.374	0.224
Hexachlorobenzene	−0.003	0.006	0.583	−0.201	0.131	0.124	−0.034	0.160	0.832	0.005	0.145	0.974	−**0.346**	**0.142**	**0.015**
β-HCH	−0.003	0.002	0.115	0.024	0.044	0.587	0.035	0.053	0.509	−**0.086**	**0.041**	**0.035**	−0.021	0.050	0.678
Dachtal	0.006	0.007	0.417	−0.183	0.151	0.227	0.054	0.182	0.768	0.273	0.153	0.075	0.055	0.182	0.763
*trans*-Chlordane	−0.016	0.009	0.057	−0.201	0.205	0.328	0.426	0.226	0.059	−0.029	0.220	0.896	0.270	0.237	0.255
*cis*-Chlordane	0.002	0.008	0.779	0.168	0.188	0.371	0.190	0.218	0.384	0.007	0.201	0.973	**0.523**	**0.191**	**0.006**
*trans*-Nonachlor	−0.005	0.009	0.538	0.004	0.198	0.984	0.341	0.217	0.116	0.077	0.207	0.710	**0.418**	**0.211**	**0.048**
p,p'-DDE	0.007	0.011	0.515	**0.634**	**0.196**	**0.001**	−0.422	0.263	0.108	−0.073	0.251	0.772	0.275	0.272	0.313
*cis*-Nonachlor	−0.017	0.015	0.263	0.351	0.351	0.318	**0.821**	**0.377**	**0.030**	−0.392	0.368	0.286	0.611	0.396	0.123
**PCBs**															
PCB-110	−0.003	0.003	0.350	0.029	0.077	0.708	−0.139	0.085	0.102	−0.022	0.081	0.783	−0.094	0.088	0.283
PCB-151	0.002	0.003	0.585	0.023	0.068	0.735	−**0.179**	**0.069**	**0.010**	−0.038	0.071	0.595	−**0.191**	**0.068**	**0.005**
PCB-135/144	0.000	0.002	0.991	0.053	0.050	0.291	−0.102	0.055	0.067	−0.056	0.053	0.284	−0.072	0.058	0.213
PCB-118	0.002	0.003	0.618	0.002	0.003	0.618	−**0.205**	**0.079**	**0.010**	−0.039	0.082	0.637	−**0.190**	**0.081**	**0.019**
PCB-132/153	0.001	0.003	0.786	0.030	0.072	0.684	−**0.162**	**0.077**	**0.035**	−0.033	0.076	0.664	−**0.156**	**0.077**	**0.043**
PCB-138/163	0.002	0.003	0.497	−0.040	0.060	0.499	−0.112	0.066	0.089	0.021	0.063	0.738	−0.107	0.066	0.108
PCB-175	0.002	0.004	0.703	0.114	0.089	0.202	−**0.225**	**0.096**	**0.019**	−0.139	0.093	0.133	−0.191	0.100	0.055
PCB-174	−0.002	0.004	0.660	0.047	0.095	0.624	−**0.217**	**0.100**	**0.030**	−0.006	0.100	0.953	−0.080	0.109	0.464
PCB-202	0.006	0.005	0.192	−0.047	0.108	0.661	−**0.258**	**0.113**	**0.022**	0.110	0.111	0.323	−0.117	0.123	0.341
PCB-180	0.001	0.003	0.696	−0.083	0.057	0.146	0.003	0.070	0.968	0.089	0.060	0.138	−0.049	0.069	0.479
PCB-170/190	0.005	0.006	0.386	0.073	0.134	0.584	−**0.334**	**0.138**	**0.016**	0.066	0.140	0.637	−0.170	0.152	0.262
PCB-198	0.002	0.005	0.681	0.007	0.103	0.943	0.073	0.118	0.539	0.051	0.107	0.634	−0.003	0.120	0.978
**BFRs**															
TBBPa	0.000	0.001	0.949	0.032	0.025	0.202	0.006	0.030	0.834	−0.035	0.026	0.186	−0.005	0.030	0.870
PBDE-28	−0.009	0.006	0.114	−0.099	0.141	0.483	0.189	0.161	0.240	0.079	0.149	0.595	0.210	0.160	0.188
PBDE-75	−0.008	0.008	0.332	0.054	0.188	0.772	0.086	0.218	0.694	−0.147	0.195	0.452	0.291	0.209	0.164
PBDE-47	−0.001	0.001	0.217	−0.030	0.045	0.505	**0.118**	**0.046**	**0.011**	−0.057	0.046	0.219	−0.007	0.053	0.901
PBDE-66	0.001	0.001	0.370	0.077	0.103	0.454	−**0.295**	**0.102**	**0.004**	0.158	0.104	0.126	0.036	0.121	0.768
PBDE-100	0.006	0.004	0.168	0.113	0.101	0.264	−0.189	0.114	0.097	0.143	0.105	0.172	0.156	0.116	0.180
PBDE-99	−0.001	0.001	0.168	−0.021	0.017	0.215	0.034	0.019	0.075	0.015	0.018	0.422	0.022	0.020	0.265
PBDE-85	0.001	0.002	0.990	0.049	0.129	0.702	−0.249	0.140	0.076	0.152	0.131	0.249	−0.094	0.149	0.526
PBDE-154	−0.021	0.012	0.075	0.273	0.283	0.334	**0.682**	**0.301**	**0.024**	−0.293	0.297	0.323	0.390	0.325	0.230
PBDE-153	0.004	0.006	0.487	**0.311**	**0.107**	**0.004**	−0.162	0.143	0.258	−0.163	0.128	0.203	−0.003	0.147	0.986

*β-HCH, beta-hexachlorocyclohexane; PCB, polychlorinated biphenyl; BFR, brominated flame retardant; TBBPA, tetrabromobisphenol A; PBDE, polybromodiphenyl ether; SE, standard error. p-value < 0.05 shown in bold type*.

Our analysis of demographic and smoking variables is exploratory and has important limitations. First, the small sample size provides little power to detect effects and does not allow generalization. Second, multiple comparisons must be considered given the five models run for the 32 POPs, i.e., there is a 19% chance (1–0.95^4^) that the null hypothesis would be falsely rejected for each POP. Third, outliers and measurements below detection limits could affect results, e.g., PBDE-66 and 85 initially were associated with age, however, the significance disappeared when measurements below detection limits were removed (Supplemental Figure S3).

### Recommendations and applications

Measurements of POPs in plasma and whole blood are widely used and represent an exposure metric that integrates across exposure pathways and sources (Sexton et al., 2004; Aylward et al., 2014). Concentrations in plasma exceed levels in whole blood and DBS, but distribution coefficients allow adjustment and comparison across studies and study types (CDC, 2015a). Distribution coefficients estimated in the current study allow comparisons across studies, including with national norms. Theoretical predictions (using Equation 1) did not fully account for differences in solubility of plasma and whole blood, thus experimentally-derived distribution coefficients should be used. For PCBs and PBDEs, the use of the average experimentally-derived plasma/whole blood coefficient should be suitable, while compound-specific values are recommended for pesticides/CHCs and tetrabromobisphenol A. The values reported in this study are reliable and have excellent reproducibility.

Most earlier studies have utilized plasma samples for POP biomarker analyses. While plasma requires the extra steps of centrifuging and then pipetting the sample (or removing the pellet), somewhat less cleanup is required, and POP concentrations are higher. However, many studies have been conducted using whole blood, and some researchers prefer to avoid the extra steps, logistical issues, and risks of contamination involved in obtaining plasma (Angerer, 2006). DBS sampling is receiving increased attention due to its ease and safety of collection, transport and storage, and the development of advanced analytical technology that permits quantitation in very small samples. DBS sampling may be cost-effective and suitable for large studies and longitudinal study designs (Jones and Golding, 2009; Ostler et al., 2014). Obtaining very low DBS detection limits is challenging given the very small sample volume available; this might be addressed with additional sample preconcentration, for example, using on-line extraction and LC/MS-MS, a technique with the promise of increasing throughput and sensitivity (Lu et al., 2012). In addition, DBS and other sample types can involve important concerns regarding storage stability and other issues that may affect their utility, particularly for archived samples used for retrospective exposure assessment (Batterman and Chernyak, 2014).

### Strengths and limitations

Distribution coefficients between plasma and whole blood were estimated for a wide range of POPs, providing information that can be used to compare POP measurements among different blood-based sample types. Very similar sample preparation and analytical methods were used for the three sample types, and most results are consistent and precise. This study had several limitations. Detection frequencies were low for several POPs and some sample types. While three broad classes of POPs were measured, there are many other compounds of interest as biomarkers. The small convenience sample is not representative of all populations, and the measured data may not capture extreme values. The sample size is too limited to provide robust estimates of factors that might affect the variation of the distribution. Only linear relationships between plasma, whole blood, and DBS samples were investigated, but data (and theory) suggest these are warranted. Finally, we did not evaluate other analytical methods that might improve sensitivity, which is particularly important for the DBS samples.

## Conclusions

Experimentally-determined human whole blood/plasma distribution coefficients K_p∕b_ ranged from 1.74 to 2.26 for pesticides/CHCs, averaged 1.69 ± 0.06 for PCBs, 1.65 ± 0.03 for PBDEs, and 1.75 for tetrabromobisphenol A. Participant sex, race and smoking status had only minor effects on these results. For most compounds, whole blood and DBS samples were very similar, e.g., thus, K_b∕d_ = 1. These coefficients provide useful information for comparing POP exposure levels across studies using different types of blood matrices. POP measurements in DBS samples correlated closely to those in the other sample types, however, the limited sample volume increased detection limits and reduced the number of compounds that could be detected in this sample type.

## Author contributions

Study concept and design: SB, SC, and FS. Acquisition, analysis, or interpretation of data: SB, SC, and FS. Drafting of the manuscript: SB, SC, and FS. Critical revision of the manuscript for important intellectual content: SB, SC, and FS. Statistical analysis: SB and FS. Obtained funding: SB. Administrative, technical, or material support: SB and SC. Study supervision: SB.

### Conflict of interest statement

The authors declare that the research was conducted in the absence of any commercial or financial relationships that could be construed as a potential conflict of interest.

## Abbreviations

BFR: Brominated flame retardant
CDS: Certified calibration standard
CHC: Chlorinated hydrocarbon
DBS: Dried blood spot
GC/MS: Gas chromatograph/mass spectrometer
GOF: Goodness-of-fit
HCH: Hexachlorocyclohexane
IDL: Instrumental detection limit
IS: Internal standard
K_ow_: Octanol-water distribution coefficient
K_p∕b_: Plasma-whole blood distribution coefficient
MTBE: Methyl-t-butyl ether
NHANES: National Health and Nutrition Examination Survey
OCP: Organochlorine pesticide
PBDE: Polybrominated diphenyl ether
PCB: Polychlorinated biphenyl
POP: Persistent organic compound
QA: Quality assurance
QC: Quality control
SRM: Standard reference material.
