# Supplementary material for: Measurement and Comparison of Organic Compound Concentrations in Plasma, Whole Blood, and Dried Blood Spot Samples
Source: Front Genet. 2016 Apr 21;7:64. doi: 10.3389/fgene.2016.00064 (PMC4838759; doi:10.3389/fgene.2016.00064)
Supplement: Supplementary file 1 [file Table1.DOCX]

**Measurement and Comparison of Organic Compound Concentrations**

**in Plasma, Whole Blood and Dried Blood Spot Samples**

Stuart Batterman*, Sergey Chernyak, Feng-Chiao Su

Department of Environmental Health Sciences

University of Michigan, Ann Arbor, MI, USA

Feb. 3, 2016

M6075 SPH II, 1415 Washington Heights, Ann Arbor, Michigan 48109-2029, USA

*Corresponding author: Tel: 734-763-2417 Fax: 734-936-7283 Email: [stuartb@umich.edu](mailto:stuartb@umich.edu)

Supplemental Table S1. Method detection limits and detection frequencies by sample type.

| Chemical | IDL (ng L^-1^) |  |  | Detection Frequency (%)* | | |
| --- | --- | --- | --- | --- | --- | --- |
|  | Plasma, Whole Blood | DBS |  | Plasma | Whole Blood | DBS |
| **Pesticides + CHC** |  |  |  |  |  |  |
| Pentachlorobenzene | 30 | 120 |  | 95.2 | 100.0 | 0.0 |
| α-HCH | 10 | 50 |  | 47.6 | 33.3 | 0.0 |
| Hexachlorobenzene | 10 | 50 |  | 95.2 | 95.2 | 90.5 |
| PCPME | 10 | 50 |  | 0.0 | 0.0 | 0.0 |
| β-HCH | 20 | 60 |  | 100.0 | 100.0 | 90.5 |
| Aldrin | 15 | 40 |  | 0.0 | 0.0 | 0.0 |
| Dachtal | 10 | 120 |  | 90.5 | 90.5 | 0.0 |
| Octachlorostyrene | 15 | 40 |  | 0.0 | 0.0 | 0.0 |
| b-Heptachlorepoxide | 15 | 40 |  | 33.3 | 23.8 | 0.0 |
| Oxychlordane | 5 | 10 |  | 23.8 | 23.8 | 0.0 |
| *trans*-Chlordane | 10 | 40 |  | 100.0 | 95.2 | 0.0 |
| *cis*-Chlordane | 10 | 45 |  | 90.5 | 85.7 | 0.0 |
| *trans*-Nonachlor | 5 | 12 |  | 95.2 | 95.2 | 57.1 |
| Dieldrin | 10 | 30 |  | 9.5 | 9.5 | 0.0 |
| p,p'-DDE | 30 | 100 |  | 76.2 | 81.0 | 76.2 |
| Endrin | 10 | 30 |  | 4.8 | 4.8 | 0.0 |
| *cis*-Nonachlor | 10 | 40 |  | 95.2 | 85.7 | 0.0 |
| p,p'-DDD | 15 | 40 |  | 0.0 | 0.0 | 0.0 |
| p,p'-DDT | 15 | 40 |  | 0.0 | 0.0 | 0.0 |
| photo-Mirex | 15 | 40 |  | 0.0 | 0.0 | 0.0 |
| Mirex | 10 | 30 |  | 9.5 | 4.8 | 0.0 |
| **PCBs** |  |  |  |  |  |  |
| PCB-110 | 0.5 | 20 |  | 100.0 | 100.0 | 0.0 |
| PCB-151 | 0.4 | 0.8 |  | 100.0 | 100.0 | 95.2 |
| PCB-135/144 | 0.6 | 14 |  | 100.0 | 100.0 | 0.0 |
| PCB-118 | 0.6 | 0.8 |  | 100.0 | 100.0 | 95.2 |
| PCB-132/153 | 0.4 | 10 |  | 100.0 | 100.0 | 100.0 |
| PCB-138/163 | 0.8 | 5 |  | 100.0 | 100.0 | 100.0 |
| PCB-175 | 0.9 | 22 |  | 100.0 | 100.0 | 0.0 |
| PCB-174 | 0.1 | 15 |  | 100.0 | 100.0 | 0.0 |
| PCB-202 | 0.1 | 5 |  | 100.0 | 100.0 | 0.0 |
| PCB-180 | 0.3 | 7 |  | 100.0 | 100.0 | 100.0 |
| PCB-170/190 | 0.8 | 8 |  | 95.2 | 95.2 | 0.0 |
| PCB-198 | 0.2 | 7 |  | 100.0 | 100.0 | 0.0 |
| **BFRs** |  |  |  |  |  |  |
| TBBPa | 4 | 12 |  | 100.0 | 100.0 | 81.0 |
| BDE-28 | 7 | 40 |  | 76.2 | 76.2 | 0.0 |
| BDE-75 | 5 | 40 |  | 52.4 | 52.4 | 0.0 |
| BDE-49 | 5 | 30 |  | 9.5 | 9.5 | 0.0 |
| BDE-71 | 1 | 30 |  | 14.3 | 14.3 | 0.0 |
| BDE-47 | 5 | 120 |  | 100.0 | 100.0 | 100.0 |
| BDE-66 | 5 | 120 |  | 81.0 | 85.7 | 0.0 |
| BDE-100 | 8 | 40 |  | 81.0 | 85.7 | 81.0 |
| BDE-99 | 1 | 20 |  | 100.0 | 100.0 | 100.0 |
| BDE-85 | 0.5 | 22 |  | 71.4 | 71.4 | 0.0 |
| BDE-154 | 0.1 | 20 |  | 95.2 | 95.2 | 95.2 |
| BDE-153 | 0.1 | 8 |  | 90.5 | 90.5 | 0.0 |
| BDE-138 | 7 | 40 |  | 9.5 | 0.0 | 0.0 |
| BDE-166 | 7 | 40 |  | 4.8 | 4.8 | 0.0 |
| BDE-183 | 7 | 40 |  | 14.3 | 9.5 | 0.0 |
| BDE-190 | 7 | 40 |  | 0.0 | 0.0 | 0.0 |
| BDE-203 | 7 | 40 |  | 0.0 | 0.0 | 0.0 |
| BDE-208 | 20 | 140 |  | 0.0 | 0.0 | 0.0 |
| BDE-207 | 20 | 140 |  | 0.0 | 0.0 | 0.0 |
| BDE-206 | 20 | 140 |  | 0.0 | 0.0 | 0.0 |
| BDE-209 | 30 | 200 |  | 4.8 | 4.8 | 4.8 |

*, chemicals with detection frequencies < 25% were excluded from further analyses.

IDL, method of detection limits; DBS, dried blood spot; CHC, chlorinated hydrocarbons; HCH, hexachlorocyclohexane; PCB, polychlorinated biphenyl; BFR, brominated flame retardant; BDE, polybrominated diphenyl ethers.

Supplemental Table S2. Demographics and smoking status for participants in this study (n = 21).

| Variable | Value |
| --- | --- |
| Age (year) |  |
| Mean ± SD | 48.2 ± 11.5 |
| Range | 29 - 65 |
| Sex |  |
| Female | 9 (42.9%) |
| Male | 12 (57.1%) |
| Race |  |
| White | 16 (76.2%) |
| Black or Asian | 5 (23.8%) |
| Education |  |
| < graduate school | 7 (33.3%) |
| ≥ graduate school | 14 (66.7%) |
| Smoking Status |  |
| Non-smoker | 16 (76.2%) |
| Former smoker | 4 (19.0%) |
| Current smoker | 1 (4.8%) |
|  |  |

Supplemental Table S3. Spearman rank-order correlation matrix for chemical concentrations in plasma samples (n = 21).

|  | **P1** | **P2** | **P3** | **P4** | **P5** | **P6** | **P7** | **P8** | **P9** | **C1** | **C2** | **C3** | **C4** | **C5** | **C6** | **C7** | **C8** | **C9** | **C10** | **C11** | **C12** | **B1** | **B2** | **B3** | **B4** | **B5** | **B6** | **B7** | **B8** | **B9** | **B10** |
| --- | --- | --- | --- | --- | --- | --- | --- | --- | --- | --- | --- | --- | --- | --- | --- | --- | --- | --- | --- | --- | --- | --- | --- | --- | --- | --- | --- | --- | --- | --- | --- |
| **P1** | 1.00 | 0.09 | 0.30 | -0.03 | 0.23 | 0.28 | 0.38 | 0.33 | -0.09 | 0.42 | -0.19 | -0.05 | -0.09 | -0.13 | -0.11 | -0.15 | -0.10 | -0.18 | -0.10 | -0.21 | -0.12 | 0.26 | -0.02 | -0.14 | 0.09 | -0.07 | -0.03 | 0.02 | -0.22 | 0.03 | 0.01 |
| **P2** |  | 1.00 | 0.19 | **0.45** | 0.08 | -0.16 | 0.28 | -0.09 | **0.44** | -0.06 | 0.26 | 0.40 | 0.22 | 0.05 | 0.17 | 0.08 | 0.29 | 0.26 | 0.38 | 0.29 | 0.40 | 0.41 | 0.11 | -0.09 | -0.04 | **0.47** | 0.35 | **0.43** | 0.22 | 0.37 | 0.35 |
| **P3** |  |  | 1.00 | 0.26 | **0.66** | **0.53** | 0.31 | **0.58** | -0.06 | **0.44** | **0.45** | 0.37 | **0.53** | **0.52** | **0.53** | 0.30 | **0.45** | 0.05 | 0.35 | **0.49** | **0.50** | 0.36 | -0.02 | 0.12 | 0.27 | 0.14 | -0.28 | -0.14 | -0.23 | 0.37 | 0.17 |
| **P4** |  |  |  | 1.00 | 0.14 | -0.03 | 0.13 | 0.08 | **0.53** | -0.04 | **0.49** | **0.48** | 0.43 | 0.25 | 0.42 | 0.30 | 0.39 | 0.34 | 0.40 | **0.49** | **0.51** | 0.08 | 0.06 | -0.39 | **0.56** | **0.48** | 0.43 | **0.51** | **0.69** | 0.34 | 0.19 |
| **P5** |  |  |  |  | 1.00 | **0.74** | **0.63** | **0.44** | -0.32 | 0.28 | 0.16 | 0.06 | 0.19 | **0.63** | **0.52** | 0.14 | 0.18 | -0.14 | 0.12 | 0.27 | 0.31 | 0.31 | -0.13 | 0.05 | 0.38 | -0.19 | **-0.52** | -0.36 | -0.28 | 0.07 | **0.45** |
| **P6** |  |  |  |  |  | 1.00 | 0.42 | 0.42 | -0.40 | **0.44** | 0.00 | -0.11 | 0.04 | 0.38 | 0.21 | 0.04 | -0.10 | -0.35 | 0.01 | 0.06 | 0.04 | -0.01 | -0.08 | 0.10 | 0.11 | -0.35 | **-0.57** | **-0.50** | -0.37 | -0.26 | 0.00 |
| **P7** |  |  |  |  |  |  | 1.00 | 0.40 | -0.10 | 0.41 | 0.08 | 0.10 | 0.01 | 0.25 | 0.15 | 0.12 | 0.06 | 0.02 | 0.16 | 0.11 | 0.25 | 0.26 | -0.02 | 0.00 | 0.11 | -0.11 | -0.29 | -0.17 | -0.10 | 0.16 | 0.27 |
| **P8** |  |  |  |  |  |  |  | 1.00 | 0.10 | 0.41 | 0.42 | 0.43 | **0.49** | **0.48** | **0.53** | **0.48** | 0.38 | 0.27 | **0.48** | 0.42 | **0.57** | -0.02 | -0.11 | 0.16 | 0.16 | 0.31 | 0.02 | 0.05 | -0.01 | **0.47** | 0.27 |
| **P9** |  |  |  |  |  |  |  |  | 1.00 | -0.11 | **0.59** | **0.73** | 0.43 | -0.08 | 0.13 | **0.46** | **0.52** | **0.79** | **0.50** | 0.42 | 0.37 | -0.19 | 0.41 | -0.01 | 0.04 | **0.66** | **0.74** | **0.84** | **0.54** | 0.40 | 0.12 |
| **C1** |  |  |  |  |  |  |  |  |  | 1.00 | 0.38 | 0.35 | 0.40 | 0.36 | 0.21 | 0.26 | 0.31 | 0.15 | 0.39 | 0.37 | 0.25 | 0.15 | 0.01 | -0.05 | 0.14 | 0.03 | -0.10 | -0.06 | -0.22 | -0.04 | -0.08 |
| **C2** |  |  |  |  |  |  |  |  |  |  | 1.00 | **0.92** | **0.92** | **0.61** | **0.71** | **0.74** | **0.91** | **0.81** | **0.88** | **0.91** | **0.73** | 0.11 | 0.26 | 0.16 | 0.40 | **0.60** | **0.43** | **0.51** | 0.32 | **0.45** | 0.29 |
| **C3** |  |  |  |  |  |  |  |  |  |  |  | 1.00 | **0.84** | **0.46** | **0.59** | **0.66** | **0.89** | **0.84** | **0.85** | **0.82** | **0.71** | 0.07 | 0.23 | -0.05 | 0.28 | **0.74** | **0.55** | **0.65** | 0.34 | **0.46** | 0.36 |
| **C4** |  |  |  |  |  |  |  |  |  |  |  |  | 1.00 | **0.72** | **0.81** | **0.79** | **0.90** | **0.71** | **0.90** | **0.93** | **0.84** | 0.29 | -0.02 | 0.13 | **0.50** | **0.62** | 0.43 | **0.44** | 0.31 | **0.54** | 0.38 |
| **C5** |  |  |  |  |  |  |  |  |  |  |  |  |  | 1.00 | **0.90** | **0.51** | **0.64** | 0.27 | **0.63** | **0.76** | **0.73** | 0.38 | -0.26 | 0.08 | **0.59** | 0.35 | 0.00 | 0.06 | 0.05 | 0.36 | **0.61** |
| **C6** |  |  |  |  |  |  |  |  |  |  |  |  |  |  | 1.00 | **0.67** | **0.77** | **0.44** | **0.74** | **0.84** | **0.81** | 0.33 | -0.19 | 0.06 | **0.64** | **0.51** | 0.24 | 0.25 | 0.25 | **0.51** | **0.56** |
| **C7** |  |  |  |  |  |  |  |  |  |  |  |  |  |  |  | 1.00 | **0.68** | **0.71** | **0.82** | **0.79** | **0.76** | 0.02 | -0.06 | 0.14 | 0.37 | 0.43 | 0.33 | 0.34 | 0.38 | **0.52** | 0.22 |
| **C8** |  |  |  |  |  |  |  |  |  |  |  |  |  |  |  |  | 1.00 | **0.79** | **0.81** | **0.86** | **0.72** | 0.31 | 0.14 | 0.08 | **0.44** | **0.68** | **0.51** | **0.54** | 0.26 | **0.45** | **0.45** |
| **C9** |  |  |  |  |  |  |  |  |  |  |  |  |  |  |  |  |  | 1.00 | **0.72** | **0.64** | **0.55** | -0.01 | 0.28 | 0.19 | 0.24 | **0.64** | **0.66** | **0.68** | 0.42 | 0.43 | 0.28 |
| **C10** |  |  |  |  |  |  |  |  |  |  |  |  |  |  |  |  |  |  | 1.00 | **0.89** | **0.85** | 0.15 | -0.01 | 0.10 | 0.33 | **0.65** | **0.48** | **0.48** | 0.34 | **0.53** | 0.33 |
| **C11** |  |  |  |  |  |  |  |  |  |  |  |  |  |  |  |  |  |  |  | 1.00 | **0.86** | 0.27 | 0.07 | -0.01 | **0.53** | **0.63** | 0.34 | **0.43** | 0.39 | **0.57** | 0.40 |
| **C12** |  |  |  |  |  |  |  |  |  |  |  |  |  |  |  |  |  |  |  |  | 1.00 | 0.29 | -0.27 | -0.01 | 0.38 | **0.63** | 0.33 | 0.36 | 0.38 | **0.72** | **0.56** |
| **B1** |  |  |  |  |  |  |  |  |  |  |  |  |  |  |  |  |  |  |  |  |  | 1.00 | -0.12 | 0.00 | 0.39 | 0.22 | 0.05 | 0.09 | -0.01 | 0.35 | **0.48** |
| **B2** |  |  |  |  |  |  |  |  |  |  |  |  |  |  |  |  |  |  |  |  |  |  | 1.00 | 0.34 | -0.13 | 0.06 | 0.15 | 0.29 | 0.13 | -0.01 | -0.27 |
| **B3** |  |  |  |  |  |  |  |  |  |  |  |  |  |  |  |  |  |  |  |  |  |  |  | 1.00 | -0.24 | -0.17 | -0.09 | -0.18 | -0.29 | 0.07 | -0.11 |
| **B4** |  |  |  |  |  |  |  |  |  |  |  |  |  |  |  |  |  |  |  |  |  |  |  |  | 1.00 | 0.32 | 0.19 | 0.28 | **0.44** | 0.38 | 0.32 |
| **B5** |  |  |  |  |  |  |  |  |  |  |  |  |  |  |  |  |  |  |  |  |  |  |  |  |  | 1.00 | **0.78** | **0.84** | **0.51** | **0.67** | 0.35 |
| **B6** |  |  |  |  |  |  |  |  |  |  |  |  |  |  |  |  |  |  |  |  |  |  |  |  |  |  | 1.00 | **0.90** | **0.63** | 0.34 | 0.17 |
| **B7** |  |  |  |  |  |  |  |  |  |  |  |  |  |  |  |  |  |  |  |  |  |  |  |  |  |  |  | 1.00 | **0.59** | **0.46** | 0.22 |
| **B8** |  |  |  |  |  |  |  |  |  |  |  |  |  |  |  |  |  |  |  |  |  |  |  |  |  |  |  |  | 1.00 | **0.47** | 0.14 |
| **B9** |  |  |  |  |  |  |  |  |  |  |  |  |  |  |  |  |  |  |  |  |  |  |  |  |  |  |  |  |  | 1.00 | 0.32 |
| **B10** |  |  |  |  |  |  |  |  |  |  |  |  |  |  |  |  |  |  |  |  |  |  |  |  |  |  |  |  |  |  | 1.00 |

Correlation coefficients with p-value < 0.05 are shown in bold type.

P1 to P9 are pentachlorobenzene, hexachlorobenzene, β-HCH, Dachtal, *trans*-chlordane, *cis*-chlordane, *trans*-nonachlor, p,p'-DDE, *cis*-Nonachlor; C1 to C12 are PCB-110, 151, 135/144, 118, 132/153, 138/163, 175, 174, 202, 180, 170/190, 198; B1 to B10 are TBBPa, BDE-28, 75, 47, 66, 100, 99, 85, 154, 153.

Supplemental Table S4. Results of linear regression models with intercepts for relationships of chemical concentrations between plasma, whole blood and DBS samples (n = 21).

| Chemicals | y = plasma, x = whole blood | | | | |  | y = plasma, x = DBS | | | | |  | y = whole blood, x = DBS | | | | |
| --- | --- | --- | --- | --- | --- | --- | --- | --- | --- | --- | --- | --- | --- | --- | --- | --- | --- |
|  | R^2^ | Intercept | | Coefficient | |  | R^2^ | Intercept | | Coefficient | |  | R^2^ | Intercept | | Coefficient | |
|  |  | Estimate | SE | Estimate | SE |  |  | Estimate | SE | Estimate | SE |  |  | Estimate | SE | Estimate | SE |
| **Pesticides + CHC** |  |  |  |  |  |  |  |  |  |  |  |  |  |  |  |  |  |
| Pentachlorobenzene | 0.77 | -41.84 | 22.34 | 2.49 | 0.32 |  | - | - | - | - | - |  | - | - | - | - | - |
| α-HCH | 0.78 | -3.01 | 2.28 | 2.13 | 0.26 |  | - | - | - | - | - |  | - | - | - | - | - |
| Hexachlorobenzene | 0.96 | 37.52 | 29.16 | 37.52 | 29.16 |  | 0.94 | 51.73 | 36.73 | 1.68 | 0.10 |  | 0.99 | 5.67 | 7.37 | 1.02 | 0.02 |
| β-HCH | 0.98 | 70.69 | 37.14 | 1.73 | 0.05 |  | 0.98 | 123.87 | 37.50 | 1.70 | 0.06 |  | 0.99 | 33.21 | 15.48 | 0.98 | 0.02 |
| Dachtal | 0.94 | -4.33 | 9.92 | 1.81 | 0.11 |  | - | - | - | - | - |  | - | - | - | - | - |
| *trans*-Chlordane | 0.89 | -1.70 | 4.00 | 2.33 | 0.19 |  | - | - | - | - | - |  | - | - | - | - | - |
| *cis*-Chlordane | 0.94 | -5.69 | 2.64 | 2.39 | 0.14 |  | - | - | - | - | - |  | - | - | - | - | - |
| *trans*-Nonachlor | 0.91 | 1.15 | 3.38 | 2.14 | 0.15 |  | 0.68 | 16.93 | 5.06 | 1.53 | 0.24 |  | 0.79 | 7.02 | 1.82 | 0.74 | 0.09 |
| p,p'-DDE | 0.96 | -17.93 | 54.88 | 1.84 | 0.08 |  | 0.93 | 11.81 | 76.85 | 1.85 | 0.12 |  | 0.95 | 19.99 | 34.48 | 1.00 | 0.05 |
| *cis*-Nonachlor | 0.89 | 9.96 | 2.53 | 1.56 | 0.13 |  | - | - | - | - | - |  | - | - | - | - | - |
| **PCBs** |  |  |  |  |  |  |  |  |  |  |  |  |  |  |  |  |  |
| PCB-110 | 0.88 | 0.16 | 0.09 | 1.43 | 0.12 |  | - | - | - | - | - |  | - | - | - | - | - |
| PCB-151 | 0.91 | 1.32 | 5.84 | 1.72 | 0.12 |  | 0.82 | 13.89 | 7.40 | 1.56 | 0.17 |  | 0.93 | 6.68 | 2.54 | 0.92 | 0.06 |
| PCB-135/144 | 0.93 | -0.12 | 1.04 | 1.72 | 0.11 |  | - | - | - | - | - |  | - | - | - | - | - |
| PCB-118 | 0.92 | 10.12 | 13.51 | 1.67 | 0.12 |  | 0.55 | 45.21 | 32.56 | 1.63 | 0.34 |  | 0.52 | 27.35 | 19.29 | 0.91 | 0.20 |
| PCB-132/153 | 0.96 | 14.34 | 6.04 | 1.55 | 0.07 |  | 0.95 | 17.02 | 6.19 | 1.58 | 0.08 |  | 1.00 | 1.68 | 0.96 | 1.02 | 0.01 |
| PCB-138/163 | 0.96 | 5.37 | 5.11 | 1.58 | 0.07 |  | 0.94 | 8.48 | 6.21 | 1.55 | 0.09 |  | 0.99 | 1.67 | 1.75 | 0.99 | 0.03 |
| PCB-175 | 0.91 | 0.51 | 1.40 | 1.65 | 0.12 |  | - | - | - | - | - |  | - | - | - | - | - |
| PCB-174 | 0.85 | 0.14 | 0.18 | 1.48 | 0.14 |  | - | - | - | - | - |  | - | - | - | - | - |
| PCB-202 | 0.89 | -0.22 | 0.19 | 1.92 | 0.16 |  | - | - | - | - | - |  | - | - | - | - | - |
| PCB-180 | 0.80 | 11.35 | 15.79 | 1.54 | 0.18 |  | 0.76 | 14.86 | 17.42 | 1.50 | 0.19 |  | 0.97 | 1.15 | 3.45 | 0.98 | 0.04 |
| PCB-170/190 | 0.90 | 1.11 | 0.68 | 1.56 | 0.12 |  | - | - | - | - | - |  | - | - | - | - | - |
| PCB-198 | 0.80 | 0.03 | 0.78 | 1.58 | 0.18 |  | - | - | - | - | - |  | - | - | - | - | - |
| **BFRs** |  |  |  |  |  |  |  |  |  |  |  |  |  |  |  |  |  |
| TBBPa | 1.00 | 4.04 | 1.21 | 1.72 | 0.01 |  | 0.95 | 36.91 | 7.80 | 1.50 | 0.08 |  | 0.95 | 19.18 | 4.58 | 0.87 | 0.05 |
| BDE-28 | 0.99 | -1.03 | 0.70 | 1.64 | 0.04 |  | - | - | - | - | - |  | - | - | - | - | - |
| BDE-75 | 0.99 | -0.56 | 2.00 | 1.69 | 0.05 |  | - | - | - | - | - |  | - | - | - | - | - |
| BDE-47 | 0.99 | 12.51 | 25.28 | 1.66 | 0.04 |  | 0.97 | 34.22 | 45.82 | 34.22 | 45.82 |  | 0.98 | 11.46 | 20.39 | 1.02 | 0.03 |
| BDE-66 | 1.00 | -3.59 | 0.96 | 1.69 | 0.01 |  | - | - | - | - | - |  | - | - | - | - | - |
| BDE-100 | 0.99 | -1.36 | 1.56 | 1.68 | 0.02 |  | 0.97 | -15.71 | 6.05 | 1.83 | 0.08 |  | 0.97 | -8.71 | 3.23 | 1.09 | 0.04 |
| BDE-99 | 1.00 | 1.13 | 1.52 | 1.64 | 0.02 |  | 0.99 | 0.28 | 3.15 | 1.71 | 0.04 |  | 0.99 | -0.49 | 1.75 | 1.04 | 0.02 |
| BDE-85 | 1.00 | -1.57 | 1.08 | 1.68 | 0.02 |  | - | - | - | - | - |  | - | - | - | - | - |
| BDE-154 | 0.92 | 21.05 | 11.60 | 1.50 | 0.10 |  | 0.90 | 17.77 | 13.57 | 1.57 | 0.12 |  | 1.00 | -3.37 | 1.68 | 1.06 | 0.01 |
| BDE-153 | 0.98 | -0.45 | 0.44 | 1.66 | 0.06 |  | - | - | - | - | - |  | - | - | - | - | - |

DBS, dried blood spot; CHC, chlorinated hydrocarbons; HCH, hexachlorocyclohexane; PCB, polychlorinated biphenyl; BFR, brominated flame retardant; BDE, polybrominated diphenyl ethers; SE, standard error.

Supplemental Table S5. Partition coefficients of the POPs between plasma and whole blood samples estimated using a biologically-based model ([Jotaki et al. 2011](#_ENREF_12)).

| Compound | S_w_ | K_ow_ | S_o_ | K_t_,_p/b_ |
| --- | --- | --- | --- | --- |
| **Pesticides + CHC** |  |  |  |  |
| Pentachlorobenzene | 8.31E-01 | 1.66E+05 | 1.38E+05 | 1.048 |
| Hexachlorobenzene | 3.44E-01 | 7.24E+05 | 2.50E+05 | 1.048 |
| β-HCH | 4.04E+00 | 1.82E+04 | 7.36E+04 | 1.049 |
| Dachtal | 1.83E+01 | 1.55E+03 | 2.83E+04 | 1.058 |
| *trans*-Chlordane | 5.60E-02 | 1.82E+06 | 1.02E+05 | 1.048 |
| *cis*-Chlordane | 5.60E-02 | 1.82E+06 | 1.02E+05 | 1.048 |
| *trans*-Nonachlor | 6.12E-03 | 2.75E+06 | 1.69E+04 | 1.048 |
| p,p'-DDE | 4.00E-02 | 1.00E+06 | 4.00E+04 | 1.048 |
| *cis*-Nonachlor | 6.12E-03 | 2.75E+06 | 1.69E+04 | 1.048 |
| **PCBs** |  | ♣ |  |  |
| PCB-110 | 2.19E-02 | 9.55E+06 | 2.09E+05 | 1.048 |
| PCB-151 | 1.64E-03 | 4.17E+07 | 6.84E+04 | 1.048 |
| PCB-135/144 | 1.64E-03 | 4.17E+07 | 6.84E+04 | 1.048 |
| PCB-118 | 2.19E-02 | 9.55E+06 | 2.09E+05 | 1.048 |
| PCB-132/153 | 1.64E-03 | 4.17E+07 | 6.84E+04 | 1.048 |
| PCB-138/163 | 1.64E-03 | 4.17E+07 | 6.84E+04 | 1.048 |
| PCB-175 | 2.84E-04 | 1.86E+08 | 5.29E+04 | 1.048 |
| PCB-174 | 2.84E-04 | 1.86E+08 | 5.29E+04 | 1.048 |
| PCB-202 | 5.00E-04 | 8.13E+08 | 4.06E+05 | 1.048 |
| PCB-180 | 2.84E-04 | 1.86E+08 | 5.29E+04 | 1.048 |
| PCB-170/190 | 2.84E-04 | 1.86E+08 | 5.29E+04 | 1.048 |
| PCB-198 | 5.00E-04 | 8.13E+08 | 4.06E+05 | 1.048 |
| **BFRs** |  |  |  |  |
| TBBPa | 3.79E-10 | 1.58E+07 | 6.01E-03 | 1.048 |
| BDE-28 | 2.64E-02 | 7.59E+05 | 2.00E+04 | 1.048 |
| BDE-75 | 1.46E-03 | 5.89E+06 | 8.60E+03 | 1.048 |
| BDE-47 | 1.46E-03 | 5.89E+06 | 8.60E+03 | 1.048 |
| BDE-66 | 1.46E-03 | 5.89E+06 | 8.60E+03 | 1.048 |
| BDE-100 | 7.86E-05 | 4.57E+07 | 3.59E+03 | 1.048 |
| BDE-99 | 7.86E-05 | 4.57E+07 | 3.59E+03 | 1.048 |
| BDE-85 | 7.86E-05 | 4.57E+07 | 3.59E+03 | 1.048 |
| BDE-154 | 4.15E-06 | 3.55E+08 | 1.47E+03 | 1.048 |
| BDE-153 | 4.15E-06 | 3.55E+08 | 1.47E+03 | 1.048 |

S_w_ = solubility of chemical in water (mg L^-1^); Kow = octanol-water partition coefficient; S_o_ = solubility of chemical in n-octanol (mg L^-1^); K_t_,_p/b_ = estimated partition coefficient between plasma and whole blood, which was calculated by (S_o_N_p_ + S_w_0.7P_p_ + S_o_0.3P_p_ + S_w_W_p_) / (S_o_N_b_ + S_w_0.7P_b_ + S_o_0.3P_b_ + S_w_W_b_), N_p_ = neutral lipid content of plasma = 0.0046, P_p_ = phospholipid content of plasma = 0.0023, W_p_ = water content of plasma = 0.93, N_b_ = neutral lipid content of blood = 0.0044, P_b_ = phospholipid content of blood = 0.0021, W_b_ = water content of blood = 0.80.

CHC, chlorinated hydrocarbons; HCH, hexachlorocyclohexane; PCB, polychlorinated biphenyl; BFR, brominated flame retardant; BDE, polybrominated diphenyl ethers

**
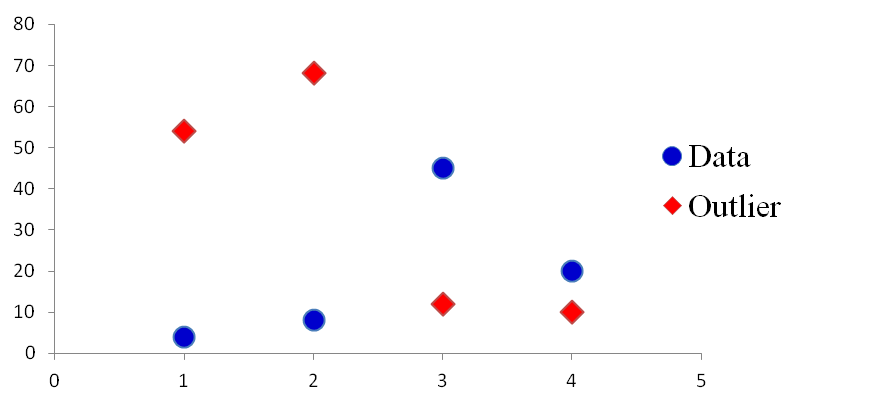
**

Plasma (ng L^-1^)

Whole blood (ng L^-1^)

Supplemental Figure S1. Relationship of pentachlorobenzene and β-hexachlorocyclohexane concentrations between plasma and whole blood samples before and after removing outliers. (A-1) and (B-1) indicates before removing the outliers for pentachlorobenzene and β-hexachlorocyclohexane, respectively, and (A-2) and (B-2) indicates after removing the outliers for pentachlorobenzene and β-hexachlorocyclohexane, respectively.

Plasma-whole blood ratio

Concentration in whole blood (ng L^-1^)

Supplemental Figure S2. Modified Bland-Altman plots for selected compounds showing relationship of plasma-whole blood ratios to whole blood concentrations for selected POPs.


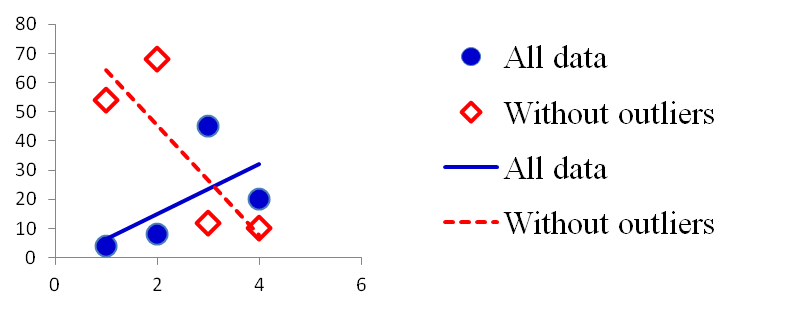


Plasma-whole blood ratio

Age (year)

Supplemental Figure S3. Relationship of plasma-whole blood ratios and age before and after removing outliers.
